# Trends and Disparities in Next-Generation Sequencing in Metastatic Prostate and Urothelial Cancers

**DOI:** 10.1001/jamanetworkopen.2024.23186

**Published:** 2024-07-18

**Authors:** Chadi Hage Chehade, Yeonjung Jo, Georges Gebrael, Nishita Tripathi, Nicolas Sayegh, Beverly Chigarira, Vinay Mathew Thomas, Gliceida Galarza Fortuna, Arshit Narang, Patrick Campbell, Sumati Gupta, Benjamin L. Maughan, Soumyajit Roy, Neeraj Agarwal, Umang Swami

**Affiliations:** 1 Division of Medical Oncology, Department of Internal Medicine, Huntsman Cancer Institute, University of Utah, Salt Lake City; 2 Division of Biostatistics, Department of Population Health Sciences, School of Medicine, University of Utah, Salt Lake City; 3 Cancer Biostatistics, Huntsman Cancer Institute, University of Utah, Salt Lake City; 4 Department of Internal Medicine, University of Texas Southwestern Medical Center, Dallas; 5 Department of Radiation Oncology, Rush University Medical Center, Chicago, Illinois

## Abstract

**Importance:**

Targeted therapies based on underlying tumor genomic susceptible alterations have been approved for patients with metastatic prostate cancer (mPC) and advanced urothelial carcinoma (aUC).

**Objective:**

To assess trends and disparities in next-generation sequencing (NGS) testing among patients with mPC and aUC.

**Design, Setting, and Participants:**

This retrospective cohort study used an electronic health record–derived database to extract deidentified data of patients receiving care from US physician practices, hospital-affiliated clinics, and academic practices. Patients diagnosed with mPC or aUC between March 1, 2015, and December 31, 2022, were included.

**Exposures:**

Social determinants of health evaluated by race and ethnicity, socioeconomic status (SES), region, insurance type, and sex (for aUC).

**Main Outcomes and Measures:**

The primary outcomes were (1) NGS testing rate by year of mPC and aUC diagnosis using Clopper-Pearson 2-sided 95% CIs and (2) time to NGS testing, which considered death as a competing risk. Cumulative incidence functions were estimated for time to NGS testing. Disparities in subdistributional incidence of NGS testing were assessed by race and ethnicity, SES, region, insurance type, and sex (for aUC) using the Fine-Gray modified Cox proportional hazards model, assuming different subdistribution baseline hazards by year of mPC and aUC diagnosis.

**Results:**

A total of 11 927 male patients with mPC (167 Asian [1.6%], 1236 Black [11.6%], 687 Hispanic or Latino [6.4%], 7037 White [66.0%], and 1535 other [14.4%] among 10 662 with known race and ethnicity) and 6490 patients with aUC (4765 male [73.4%]; 80 Asian [1.4%], 283 Black [4.8%], 257 Hispanic or Latino [4.4%], 4376 White [74.9%], and 845 other [14.5%] among 5841 with known race and ethnicity) were eligible and included. Both cohorts had a median age of 73 years (IQR, 66-80 years), and most underwent NGS testing before first-line treatment in the mPC cohort (1502 [43.0%]) and before second-line treatment in the aUC cohort (1067 [51.3%]). In the mPC cohort, the rates of NGS testing increased from 19.0% in 2015 to 27.1% in 2022, but Black patients (hazard ratio [HR], 0.75; 95% CI, 0.67-0.84) and Hispanic or Latino patients (HR, 0.70; 95% CI, 0.60-0.82) were less likely to undergo NGS testing. Patients with mPC who had low SES (quintile 1: HR, 0.74 [95% CI, 0.66-0.83]; quintile 2: HR, 0.89 [95% CI, 0.80-0.99]), had Medicaid (HR, 0.53; 95% CI, 0.38-0.74) or Medicare or other government insurance (HR, 0.89; 95% CI, 0.82-0.98), or lived in the West (HR, 0.81; 95% CI, 0.70-0.94) also were less likely to undergo testing. In the aUC cohort, the NGS rate increased from 14.1% in 2015 to 46.6% in 2022, but Black patients (HR, 0.76; 95% CI, 0.61-0.96) and those with low SES (quintile 1: HR 0.77 [95% CI, 0.66-0.89]; quintile 2: HR, 0.87 [95% CI, 0.76-1.00]) or Medicaid (HR, 0.72; 95% CI, 0.53-0.97) or Medicare or other government insurance (HR, 0.88; 95% CI, 0.78-0.99) were less likely to undergo NGS testing. Patients with aUC living in the South were more likely to undergo testing (HR, 1.29; 95% CI, 1.12-1.49).

**Conclusions and Relevance:**

These findings suggest that although NGS tumor testing rates improved over time, the majority of patients still did not undergo testing. These data may help with understanding current disparities associated with NGS testing and improving access to standard-of-care health care services.

## Key Points

**Question:**

What is the association between social determinants of health and rates of next-generation sequencing (NGS) in patients with metastatic prostate or urothelial cancer?

**Findings:**

In this cohort study of 11 927 patients with metastatic prostate cancer and 6490 patients with advanced urothelial carcinoma, NGS rates increased over time. Black race, low socioeconomic status, and Medicaid and Medicare insurance coverage were associated with lower NGS rates in both cohorts.

**Meaning:**

These findings suggest that despite the presence of actionable susceptible alterations in prostate and urothelial cancers, the majority of patients still do not undergo NGS, stressing the need to improve access to quality health care.

## Teaser

This cohort study assesses trends and disparities in next-generation sequencing testing among US patients with metastatic prostate cancer and advanced urothelial carcinoma.

## Introduction

Comprehensive genomic profiling of prostate cancer using next-generation sequencing (NGS) has defined a new era of personalized approaches in the treatment of metastatic prostate cancer (mPC).^1,2,3,4,5^ This tool has allowed clinicians to detect actionable alterations associated with improved survival outcomes with specific therapies. For instance, the presence of *BRCA* or other homologous recombination repair (HRR) alterations, found in approximately 30% of patients with advanced prostate cancer,^6^ makes patients eligible for poly (ADP-ribose) polymerase inhibitor (PARPi) monotherapy with olaparib or rucaparib or PARPi-based combinations, such as olaparib with abiraterone, niraparib with abiraterone, and talazoparib with enzalutamide, in the metastatic castration-resistant prostate cancer (mCRPC) setting.^7,8,9^ Furthermore, in patients with metastatic hormone-sensitive prostate cancer (mHSPC), the presence of *SPOP*-susceptible alterations was associated with improved outcomes in patients receiving androgen deprivation therapy intensification with an androgen receptor pathway inhibitor.^10^

In advanced urothelial carcinoma (aUC), genomic biomarkers are routinely used in treatment selection.^11^ For example, erdafitinib is approved for patients with locally advanced or metastatic urothelial carcinoma (la/mUC) harboring *FGFR3* alterations with disease progression on at least 1 line of prior systemic therapy.^12^ Moreover, pembrolizumab and dostarlimab, both programmed cell death protein 1 inhibitors, have tumor-agnostic approval for patients with solid tumors, including prostate and bladder cancer, displaying high microsatellite instability or mismatch repair deficiency.^13,14,15,16,17,18^ Pembrolizumab is also approved for patients with high tumor mutational burden (≥10 alterations/megabase) detected on NGS.^19^

Despite substantial survival improvement associated with these targeted therapies, access to NGS testing is subject to disparities. For example, in a large nationwide database, only 10.4% of patients with various tumors undergoing testing had African ancestry, 9.1% had Hispanic ancestry, and 3.7% had East Asian ancestry.^20^ In patients with mCRPC, a recent report showed that only 37.7% received HRR alteration testing, and those with low socioeconomic status (SES), covered by Medicaid insurance, or treated in a physician practice or hospital-based clinic were less likely to be tested.^21^

With the recent therapeutic advances occurring in mPC and aUC, we analyzed the current trends in NGS tumor testing in patients with mPC and aUC and assessed the association of social determinants of health with access to NGS testing in a large dataset. We hypothesized that NGS testing rates would progressively increase following the approval of susceptible alteration–targeting therapies and that disparities may exist in testing based on certain patient demographics and SES.

## Methods

### Patient Selection

This cohort study was approved by the institutional review board at the University of Utah. For the study, informed consent was waived due to the use of deidentified data. The study fully complied with the US patient confidentiality regulations, including adherence to the Health Insurance Portability and Accountability Act of 1996. The study adheres to the Strengthening the Reporting of Observational Studies in Epidemiology (STROBE) reporting guideline.

We retrospectively extracted patient-level data using a nationwide (US-based) Flatiron Health electronic health record (EHR)–derived database. This longitudinal database comprises nationally representative data mostly from physician practice and hospital-based clinic settings from 2011 through the present. This database includes structured and unstructured data curated via technology-enabled abstraction and supplemented with third-party death information. The data are deidentified and subject to obligations to prevent reidentification and protect patient confidentiality. Comparisons of the Flatiron Health database with other databases have been previously reported.^22,23^ During the study period, the data originated from approximately 280 cancer clinics (approximately 800 sites of care).

The analytic cohort included patients diagnosed with mPC or aUC between March 1, 2015, and December 31, 2022, with a data cutoff of January 31, 2023. Patients who did not receive any lines of therapy were excluded.

### Patient Exposures

Next-generation sequencing testing was performed on tumor tissue, blood, or saliva from patients with mPC and on tumor tissue or blood from patients with aUC. The NGS testing date was considered the date when patients received test results.

Social determinants of health were evaluated by race and ethnicity, which included Asian non-Hispanic, Black non-Hispanic, Hispanic or Latino, White non-Hispanic, and other (non-Hispanic Alaska Native, American Indian, Native Hawaiian, or Pacific Islander or multiracial); SES; region (Midwest, Northeast, South, or West); insurance plan (commercial, Medicare or other government program, Medicaid, or others); and sex (for patients with aUC).

Race and ethnicity were collected from deidentified EHR data, wherein clinical teams input this information. These data are typically self-reported by patients through intake interviews and forms, with variations observed among practices. The area-level SES index was determined using census block group data from the American Community Survey (2015-2019), using the Yost Index methodology.^24^ This index integrates various socioeconomic indicators, including income, property values, rental expenses, poverty rates, employment distribution, unemployment rates, and educational attainment.^24^ The Yost Index has demonstrated superior performance compared with alternative indexes in terms of area stratification and cancer inequity detection.^25^ Using the most recent documented patient residential address, population-standardized SES quintiles were applied, ranging from 1 (areas with lowest SES) to 5 (areas with highest SES).^25^

### Statistical Analysis

We aimed to assess trends and disparities in NGS testing in mPC and aUC by race and ethnicity, SES, insurance type, region, and sex (for aUC only). A trend of NGS testing rate was summarized by year of mPC or aUC diagnosis using percentages and Clopper-Pearson 95% CIs.^26^ We used a competing risk framework to estimate the incidence of NGS testing. In the time-to-NGS testing outcome, NGS testing was the main event, death was a competing risk, and loss to follow-up was a censoring event. We considered the loss to follow-up date as the patient’s last visit to the clinic or treatment end date. We estimated the cumulative incidence functions for NGS testing by exposures and compared them using the Gray test.^27^ We estimated the subdistribution hazard ratio (HR) using the Fine-Gray Cox proportional hazards model^28,29^ based on our exposures of interest (race and ethnicity, SES, region, insurance type, and sex), assuming different subdistribution baseline hazards by the year of mPC or aUC diagnosis. We performed univariable analyses for race and ethnicity, SES, and insurance type and multivariable analyses for regions adjusting for race and ethnicity and sex (only for aUC). We performed subgroup analyses by race and ethnicity to investigate the association of SES with the incidence of NGS in different racial groups. Proportional hazard assumptions were tested using Schoenfeld residuals.^30^ A 2-sided *P* < .05 was used as the threshold of statistical significance. In our analysis, subdistribution HRs indicate the likelihood of undergoing NGS testing; thus, an HR greater than 1 was considered beneficial. All the analyses were done using R, version 4.2.3 software (R Project for Statistical Computing).

## Results

Overall, 11 927 patients with mPC and 6490 patients with aUC were eligible and included. Both cohorts had a median age of 73 years (IQR, 66-80 years). Most patients received NGS testing before first-line treatment in the mPC cohort (1502 of 3489 [43.0%]) and before second-line treatment in the aUC cohort (1067 of 2079 [51.3%]). Among patients with aUC, 4765 (73.4%) were male, 1724 (26.6%) were female, and 1 (<0.1%) was of unknown sex. Race and ethnicity data were available for 10 662 patients with mPC (Asian, 167 [1.6%]; Black, 1236 [11.6%]; Hispanic or Latino, 687 [6.4%]; White, 7037 [66.0%]; other, 1535 [14.4%]) and 5841 patients with aUC (Asian, 80 [1.4%]; Black, 283 [4.8%]; Hispanic or Latino, 257 [4.4%]; White, 4376 [74.9%]; other, 845 [14.5%]). Other baseline characteristics are summarized in the Table.

**Table.** Baseline Characteristics of Patients With Metastatic Prostate Cancer and Advanced Urothelial Carcinoma

Variable	No. of patients (%)	
	Metastatic prostate cancer (n = 11 927)	Advanced urothelial carcinoma (n = 6490)
Age at diagnosis, median (IQR), y	73 (66-80)	73 (66-80)
Sex		
Female	NA	1724 (26.6)
Male	11 927 (100)	4765 (73.4)
Unknown	NA	1 (<0.1)
No. of therapies prior to NGS testing^a^		
0	1502 (43.0)	574 (27.6)
1	943 (27.0)	1067 (51.3)
2	501 (14.4)	311 (15.0)
3	309 (8.9)	89 (4.3)
4	138 (4.0)	26 (1.3)
≥5	96 (2.8)	12 (0.6)
Missing, No.	8438	4411
Race and ethnicity^a^		
Asian	167 (1.6)	80 (1.4)
Black	1236 (11.6)	283 (4.8)
Hispanic or Latino	687 (6.4)	257 (4.4)
White	7037 (66.0)	4376 (74.9)
Other^b^	1535 (14.4)	845 (14.5)
Missing, No.	1265	649
Socioeconomic status, quintile^a^		
5 (Highest)	2271 (21.0)	1173 (20.1)
4	2580 (23.9)	1425 (24.4)
3	2310 (21.4)	1238 (21.2)
2	1983 (18.3)	1134 (19.4)
1 (Lowest)	1663 (15.4)	880 (15.0)
Missing, No.	1120	640
Region^a^		
Midwest	1287 (13.2)	786 (14.7)
Northeast	1710 (17.6)	869 (16.3)
South	4982 (51.1)	2882 (54.0)
West	1763 (18.1)	799 (15.0)
Missing, No.	2185	1154
Insurance^a^		
Commercial health plan	5755 (64.9)	3486 (67.4)
Medicare or other government program	2117 (23.9)	1068 (20.6)
Medicaid	192 (2.2)	143 (2.8)
Others	800 (9.0)	475 (9.2)
Missing, No.	3063	1318
NGS testing source^c^		
Tissue	1399 (40.1)	1504 (72.3)
Blood	1365 (39.1)	332 (16.0)
Saliva	7 (0.2)	NA
Tissue and blood	80 (2.3)	4 (0.2)
Missing	638 (18.3)	239 (11.5)

Abbreviations: NA, not applicable; NGS, next-generation sequencing.

^a^ Percentages were calculated based on the number of patients with available exposure data.

^b^ Other includes non-Hispanic Alaska Native, American Indian, Native Hawaiian, or Pacific Islander or multiracial.

^c^ Percentages were calculated based on the number of patients who received NGS testing (3489 for metastatic prostate cancer and 2079 for advanced urothelial carcinoma).

In the mPC cohort, 29.3% (95% CI, 28.4%-30.1%) underwent NGS testing, and the median time to receive testing was 13.2 months (IQR, 2.6-27.6 months) from the metastatic disease diagnosis. In the aUC cohort, 32% (95% CI, 30.8%-33.2%) underwent NGS testing, and the median time to receive testing was 2.7 months (IQR, 1.2-8.6 months) after the advanced disease diagnosis.

### Patients With mPC

**Figure 1.**
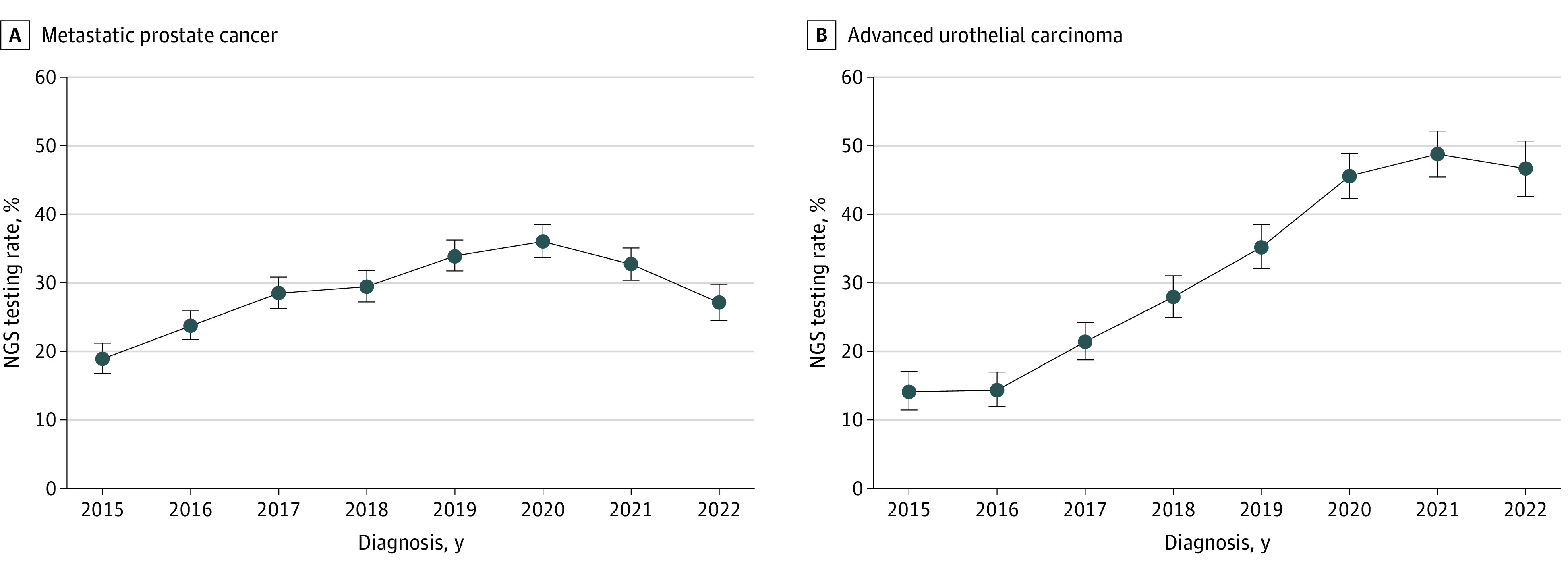
Rates of Next-Generation Sequencing (NGS) Testing by Year, 2015-2022 Whiskers indicate the 95% CI.

**Figure 2.**
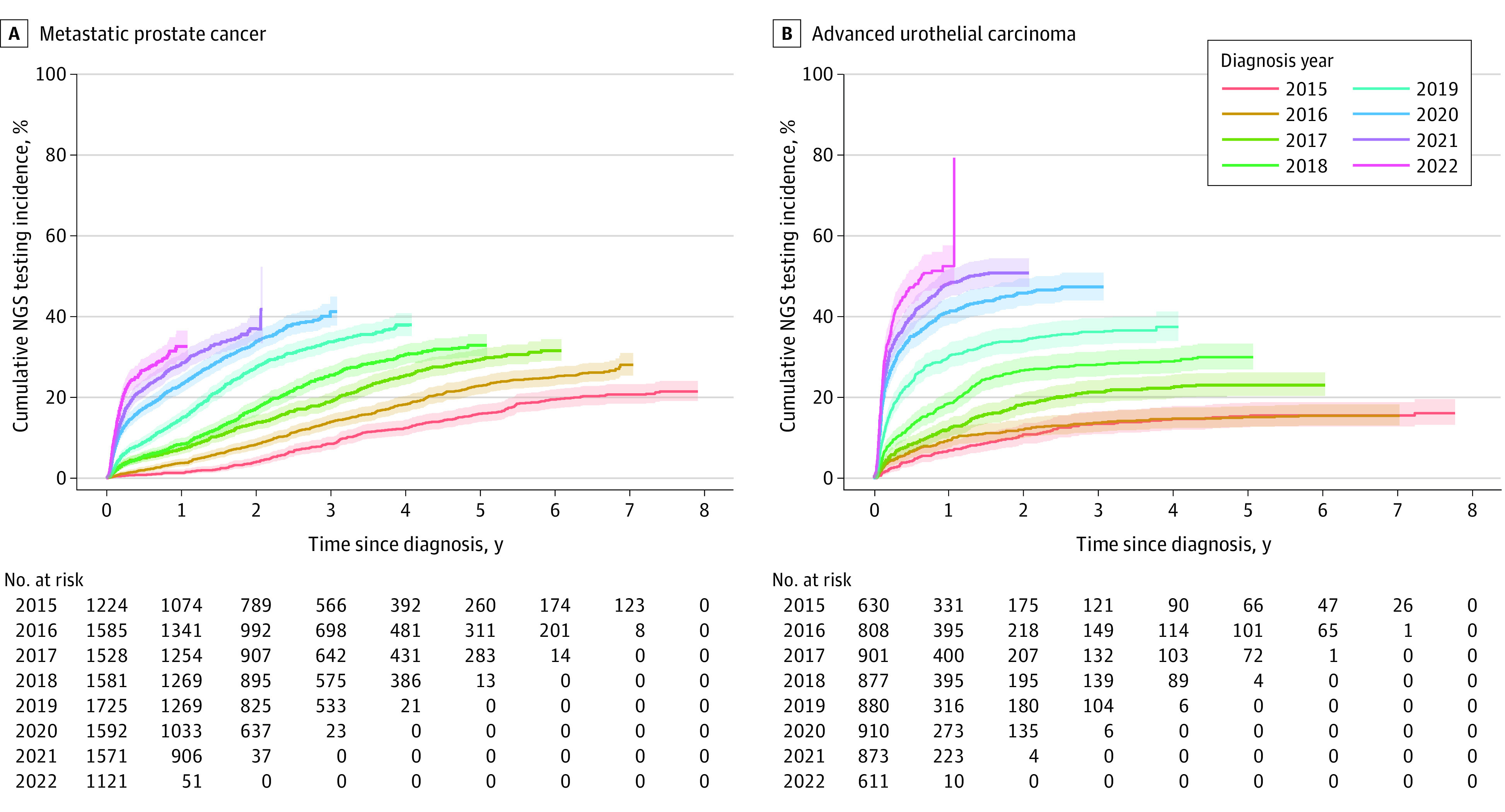
Competing Risk Analysis and Cumulative Incidence Function in Patients Undergoing Next-Generation Sequencing (NGS) Testing, 2015-2022 Shading represents the 95% CI.

Trends in NGS testing among patients with mPC are shown in Figure 1A. The rate of testing among patients in 2015 was 19.0% (95% CI, 16.8%-21.3%). This rate steadily increased until 2020, reaching 36.1% (95% CI, 33.7%-38.5%) before decreasing to 27.1% (95% CI, 24.5%-29.8%) in 2022. Figure 2A also shows an increasing trend in cumulative incidence of NGS testing in patients with mPC across all years. The cumulative incidence of NGS testing at 1 year after mPC diagnosis was 1.3% (95% CI, 1.3% to 1.3%) in 2015 and increased to 32.7% (95% CI, 32.6%-32.8%) in 2022 (*P* < .001 by Gray test) (eTable 1 in Supplement 1).

### Disparities in NGS Testing in Patients With mPC

**Figure 3.**
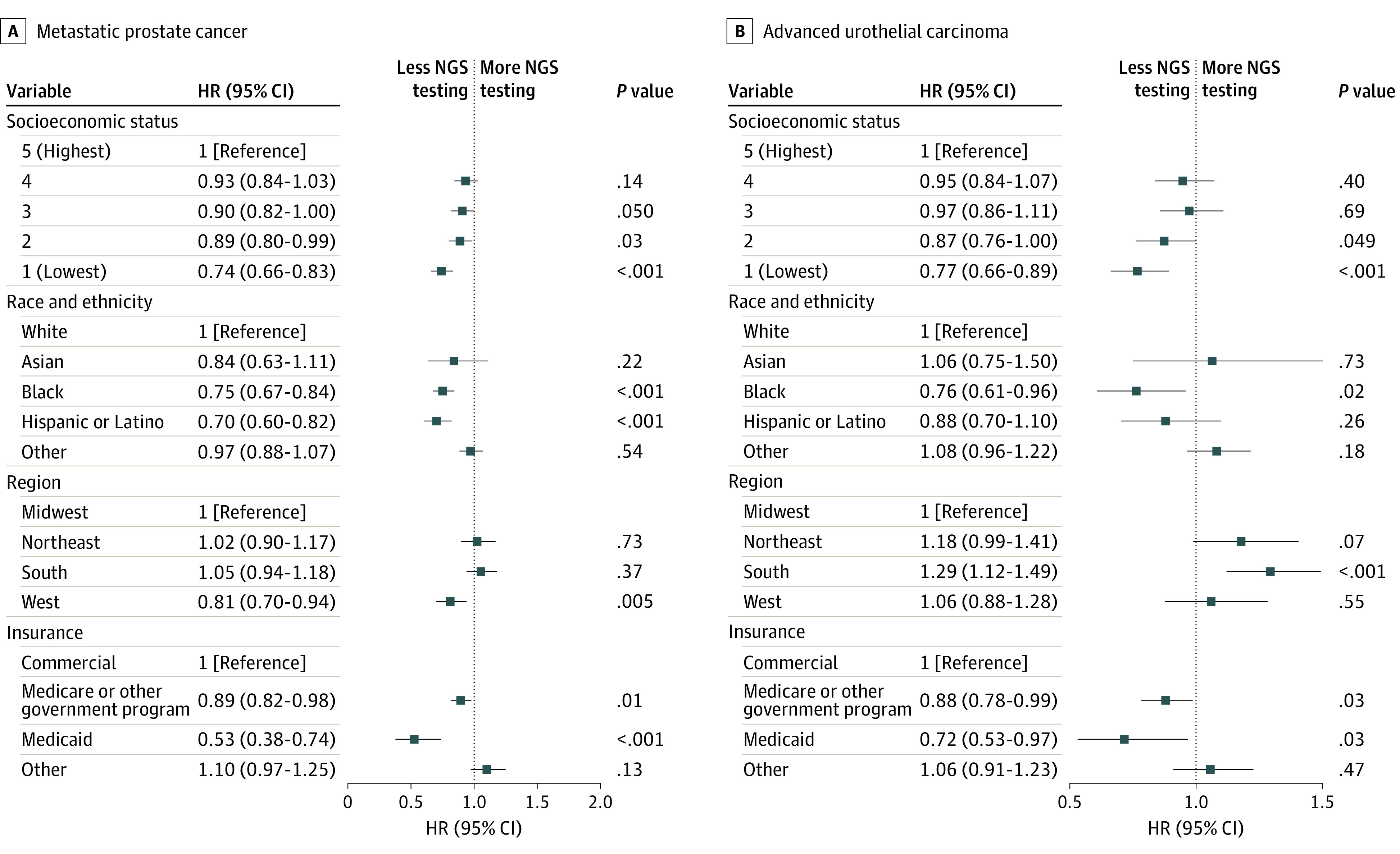
Association of Various Social Determinants of Health With the Probability of Undergoing Next-Generation Sequencing (NGS) Testing Other race and ethnicity includes non-Hispanic Alaska Native, American Indian, Native Hawaiian or Pacific Islander, or multiracial. HR indicates hazard ratio.

**Figure 4.**
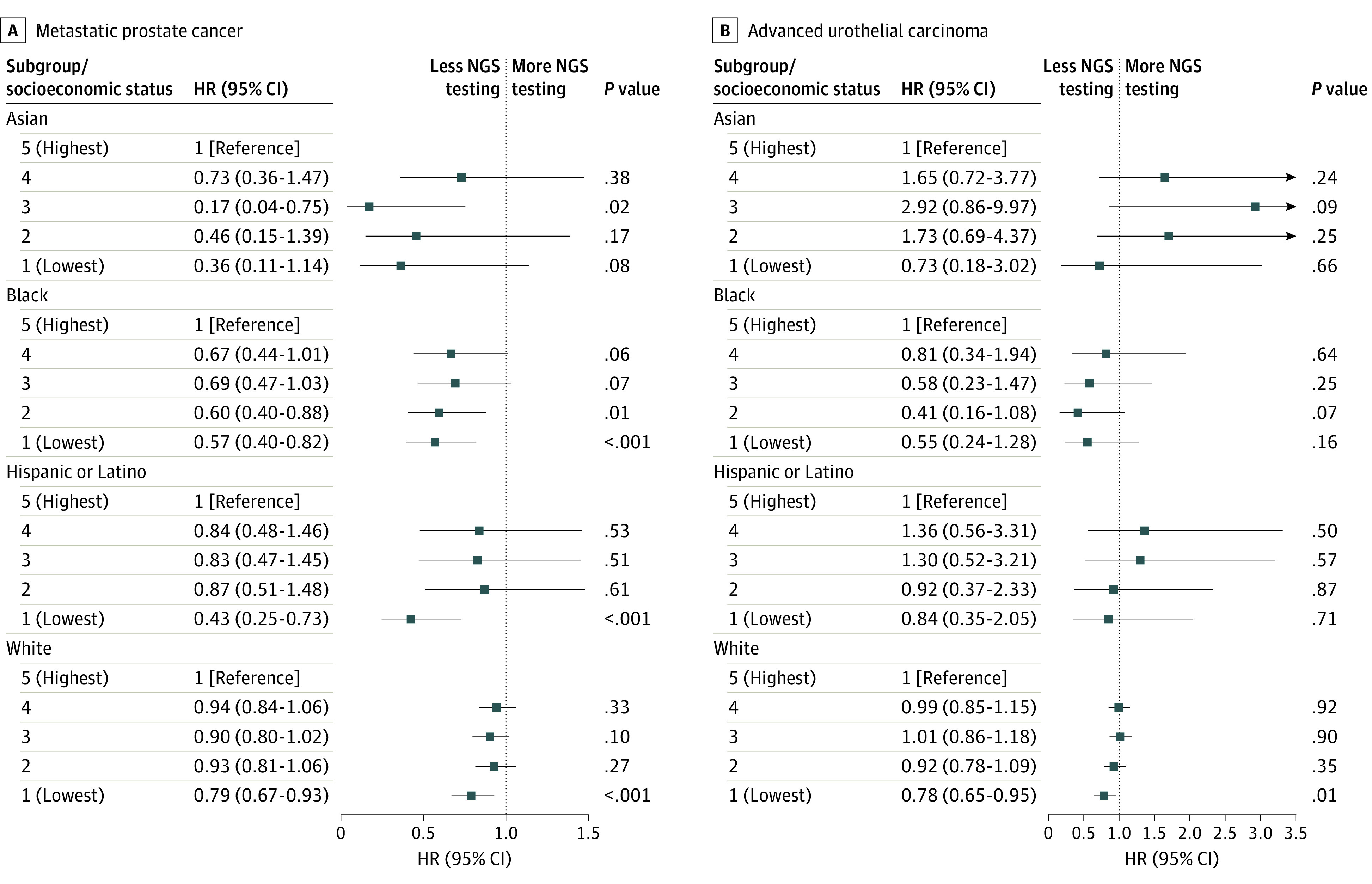
Subgroup Analysis by Race and Ethnicity of the Association of Socioeconomic Status With Next-Generation Sequencing (NGS) Tumor Testing HR indicates hazard ratio.

Compared with White patients, Black patients were significantly less likely to undergo NGS testing (HR, 0.75; 95% CI, 0.67-0.84; *P* < .001) as were Hispanic or Latino patients (HR, 0.70; 95% CI, 0.60-0.82; *P* < .001). Patients with a low SES (quintile 1: HR, 0.74 [95% CI, 0.66-0.83; *P* < .001]; quintile 2: HR, 0.89 [95% CI, 0.80-0.99; *P* = .03]) were significantly less likely to be tested than patients with the highest SES. Patients living in the West were significantly less likely to undergo NGS testing than those living in the Midwest (HR, 0.81; 95% CI, 0.70-0.94; *P* = .005), while patients with Medicare or other government insurance (HR, 0.89; 95% CI, 0.82-0.98, *P* = .01) and Medicaid (HR, 0.53; 95% CI, 0.38-0.74; *P* < .001) were significantly less likely to undergo NGS testing than those with a commercial health plan (Figure 3A). Subgroup analyses based on race and ethnicity showed that the lowest SES (ie, quintile 1) remained significantly associated with a lower likelihood of undergoing NGS testing among Black (HR, 0.57; 95% CI, 0.40-0.82; *P* < .001), Hispanic or Latino (HR, 0.43; 95% CI, 0.25-0.73; *P* < .001), and White (HR, 0.79; 95% CI, 0.67-0.93; *P* < .001) patients (Figure 4A). Additional cumulative incidence functions for NGS testing by various exposures (ie, race and ethnicity, SES, region, insurance type, and sex) after mPC diagnosis are reported in eFigures 1 to 5 in Supplement 1.

### Patients With aUC

Trends in NGS testing among patients with aUC are shown in Figure 1B. The rate of testing among patients in 2015 was 14.1% (95% CI, 11.5%-17.1%). By 2021, the rates had increased, reaching 48.8% (95% CI, 45.4%-52.2%) before slightly decreasing to 46.6% (95% CI, 42.6%-50.7%) in 2022. Figure 2B also shows an improving trend in cumulative incidence of NGS testing in patients with aUC across all years. The cumulative incidence of NGS testing at 1 year after aUC diagnosis was 6.9% (95% CI, 6.9%-6.9%) in 2015 and increased to 52.5% (95% CI, 52.4%-52.6%) in 2022 (*P* < .001 by Gray test) (eTable 2 in Supplement 1).

### Disparities in NGS Testing in Patients With aUC

Black patients were significantly less likely to undergo NGS testing than White patients (HR, 0.76; 95% CI, 0.61-0.96; *P* = .02). Compared with patients with high SES, those with low SES (quintile 1: HR, 0.77 [95% CI, 0.66-0.89; *P* < .001]; quintile 2: HR, 0.87 [95% CI, 0.76-1.00; *P* = .049]) were less likely to undergo NGS testing. Patients living in the South underwent significantly more NGS testing than those living in the Midwest (HR, 1.29; 95% CI, 1.12-1.49; *P* < .001). Patients covered by Medicare or other government insurance (HR, 0.88; 95% CI, 0.78-0.99; *P* = .03) and Medicaid (HR, 0.72; 95% CI, 0.53-0.97; *P* = .03) had significantly less testing than those with a commercial health plan (Figure 3B). Subgroup analyses based on race and ethnicity showed that the lowest SES (ie, quintile 1) remained significantly associated with a lower likelihood of testing only among White patients (HR, 0.78; 95% CI, 0.65-0.95; *P* = .01) (Figure 4B). Additional cumulative incidence functions for NGS testing by various exposures (ie, race and ethnicity, SES, region, insurance type, and sex) after aUC diagnosis are reported in eFigures 1 to 5 in Supplement 1.

## Discussion

The findings of this large cohort study assessing trends and disparities in NGS tumor testing among US patients with mPC and aUC show that while the rates of NGS have improved over time, the majority of patients in both cohorts did not undergo testing. Furthermore, patients with mPC who were Black or Hispanic or Latino; resided in the West; had a low SES; or had Medicaid, Medicare, or other government insurance coverage were less likely to undergo NGS testing. Similarly, patients with aUC who were Black; had low SES; or had Medicaid, Medicare, or other government insurance coverage were less likely to be tested but more likely if they lived in the South.

In patients with mPC, the rate of NGS increased from 19.0% in 2015 to 27.1% in 2022. This low rate in 2022 aligns with previous data suggesting a low testing rate in patients with prostate cancer, who were 10 times less likely to undergo NGS testing than patients with lung cancer and 4 times less likely than those with colorectal cancer.^31^ In another study, only 10.4% of patients with mPC underwent testing within 30 days of the metastatic disease diagnosis.^32^ However, it is important to acknowledge that therapies targeting tumor susceptible alterations, such as PARPis or pembrolizumab, were approved for mPC in 2020 and 2017, respectively, which may explain the low testing rate encountered in our cohort in 2015 and 2016 before the PARPi approval and the increase after 2020 (Figure 2A). The PARPis have substantially improved survival outcomes in patients with mCRPC. For instance, in the PROfound (Olaparib [Lynparza] Versus Enzalutamide or Abiraterone Acetate in Men With mCRPC) trial, patients harboring HRR alterations who received olaparib had a 51% reduction in the risk of radiographic progression or death compared with those treated with the physician’s choice of enzalutamide or abiraterone.^6^ Similarly, in the TALAPRO-2 (Talazoparib Plus Enzalutamide Versus Enzalutamide Monotherapy in mCRPC) trial, patients with HRR alterations treated with talazoparib and enzalutamide had a 54% reduction in the risk of radiographic progression or death compared with those treated with enzalutamide alone.^7^ It is important to highlight that delays in NGS testing of tumors may lead to difficulties with NGS assessment due to loss of tumor tissue quantity and quality over time. For example, in the PROfound trial, one-third of patients could not enroll due to unsuccessful sequencing.^6^

Besides the role of NGS testing in treatment selection, it may also assist in prognostication and patient counseling by uncovering susceptible alterations potentially associated with worse survival outcomes. For instance, previous studies have shown that alterations in tumor suppressor genes, including *RB1*, *PTEN*, and *TP53*, could be associated with more aggressive disease features and worse survival outcomes, and the presence of these genetic susceptible alterations may warrant more frequent monitoring with imaging studies rather than surveillance of serum prostate-specific antigen levels.^2,33,34,35,36,37^ Furthermore, ongoing trials are investigating additional susceptible alteration–targeting agents in an earlier disease setting. TALAPRO-3 (Talazoparib With Enzalutamide in Men With DDR Gene Mutated mCSPC)^38^ and AMPLITUDE (Niraparib in Combination With Abiraterone Acetate and Prednisone Versus Abiraterone Acetate and Prednisone for the Treatment of Participants With Deleterious Germline or Somatic HRR Gene-Mutated mCSPC)^39^ are assessing the combinations of talazoparib with enzalutamide and niraparib with abiraterone, respectively, in patients with mHSPC harboring deleterious HRR alterations. CAPItello-281 (Capivasertib Plus Abiraterone as Treatment for Patients With mHSPC and PTEN Deficiency)^40^ also compares capivasertib (an *AKT* inhibitor) with abiraterone vs abiraterone in patients with *PTEN* deficiency receiving androgen deprivation therapy in the mHSPC de novo setting.

In patients with aUC, the rate of NGS testing also increased from 14.1% in 2015 to 46.6% in 2022. Despite these improvements, testing in urothelial carcinoma is still lacking compared with other solid tumors since these patients were approximately 5 times less likely to be tested than those with lung cancer and 2 times less likely than those with colorectal cancer.^31^ Tumor NGS testing has acquired growing importance in urothelial carcinoma after the approval of susceptible alteration–targeting drugs. Erdafitinib was granted accelerated US Food and Drug Administration approval in 2019, which may help to explain the important increase in NGS testing between 2018 and 2020 and the low testing rate in 2015 before the approval of erdafitinib (Figure 2B). Patients with la/mUC presenting *FGFR2*/*3* alterations with prior progression on anti–programmed cell death protein 1 or anti–programmed cell death ligand-1 agents had a 36% reduction in the risk of death when treated with erdafitinib compared with chemotherapy.^41^ Based on these results, the erdafitinib Food and Drug Administration label was updated in 2024 to include patients with la/mUC with susceptible *FGFR3* alterations and progression on 1 prior systemic therapy.^12^

Racial and ethnic disparities remain an important issue in prostate and urothelial cancers. For example, Black patients are 2 times more likely to die of prostate cancer compared with White patients.^42^ However, another study in the context of mHSPC showed that in patients enrolled in a clinical trial, the survival outcomes of Black compared with White patients were similar, underscoring the importance of addressing disparities in access to high-quality health care.^43^

In urothelial carcinoma, a nationwide study found that Black patients were more likely to be diagnosed with more advanced disease stages.^44^ In our study, we found that Black patients were less likely to undergo NGS in both the mPC and aUC cohorts, and Hispanic or Latino patients were less likely to undergo NGS in the mPC cohort. These findings align with previous studies showing that Black patients underwent less genomic testing for non–small-cell lung cancer and colorectal carcinoma in the US compared with White patients.^45^ There remains a crucial need to alleviate these racial disparities, including in genomic testing. The plausible explanations for the decreased rate of NGS testing may be limited comprehension of the terminology and process, distrust of the medical system, hesitancy in seeking health care, and a scarcity of genetic counselors coupled with deficiencies in existing genetic counseling models that disproportionately affect racial minorities.^46^

Furthermore, patients with low SES and prostate and urothelial cancers appear to have substantially decreased survival rates.^47,48^ Our study also shows that these patients were less likely to undergo NGS than patients with higher SES. In 2018, Medicare released a National Coverage Determination (NCD) memorandum that categorized NGS as an essential diagnostic tool for patients with advanced or metastatic cancer,^49^ which may help to improve access to NGS testing for patients with low SES. Similarly, this policy may explain the increase in the rate of NGS testing after 2018 that we observed in both the aUC and mPC cohorts. In fact, in a study assessing the trends in NGS testing for patients with non–small-cell lung cancer, colorectal cancer, breast cancer, or melanoma before and after the release of the NCD, the rate of NGS testing increased post NCD across all insurance plans.^50^

To our knowledge, our study is the largest study to date to assess trends and disparities in patients with mPC and patients with aUC. We relied on a nationwide patient-level database representative of the US population spanning a period of 7 years to analyze the annual changes in NGS in these cohorts. Our findings reveal an underrepresentation of specific patient demographics in tumor genomic profiling, indicating disparities in health care delivery. These results underscore the imperative for initiatives aimed at bridging these gaps.

### Limitations

The limitations of our study include its retrospective nature and data missingness in certain patient exposures since we relied on EHR reporting. Our sample is also not homogenous in terms of geographic representation since most of the patients resided in the South. Furthermore, this study does not differentiate between patients who underwent somatic vs germline NGS testing. All exposures were measured at baseline (ie, mPC or aUC diagnosis date), and potential biases (such as access to genetic counseling) and changes over time in patient characteristics (such as SES, region, and insurance) could not be controlled. We also adjusted for changes in NGS recommendations by assuming different baseline hazards depending on the year of mPC or aUC diagnosis; however, clustering by practice type and other factors may also be present. Furthermore, since our data cutoff was January 31, 2023, there may have been an underestimation of NGS rates for patients receiving their diagnosis toward the end of 2022.

## Conclusions

The findings of this cohort study suggest that while the rate of NGS improved over time, the majority of patients with mPC and aUC still did not undergo NGS testing. Social determinants of health, such as race and ethnicity, SES, and insurance type, may be associated with access to NGS testing. Upon external validation, these hypothesis-generating data may help with understanding current disparities associated with NGS testing and improve access to standard-of-care approaches and therapies by shaping health care policies.

## Notes

Supplement 1. **eTable 1.** Cumulative Incidence at 6 Months, 1 Year, 2 Years, and 3 Years by Year of Diagnosis and Different Exposures (Race/Ethnicity, Socioeconomic Status, Region, and Insurance) in Patients With Metastatic Prostate Cancer **eTable 2.** Cumulative Incidence at 6 Months, 1 Year, 2 Years, and 3 Years by Year of Diagnosis and Different Exposures (Race/Ethnicity, Socioeconomic Status, Region, Insurance, and Sex) in Patients With Advanced Urothelial Carcinoma **eFigure 1.** Cumulative Incidence Function by Race/Ethnicity in Patients With Metastatic Prostate Cancer (A) and Advanced Urothelial Carcinoma (B) **eFigure 2.** Cumulative Incidence Function by Socioeconomic Status In Patients With Metastatic Prostate Cancer (A) and Advanced Urothelial Carcinoma (B) **eFigure 3.** Cumulative Incidence Function by Region in Patients With Metastatic Prostate Cancer (A) and Advanced Urothelial Carcinoma (B) **eFigure 4.** Cumulative Incidence Function by Insurance Plan in Patients With Metastatic Prostate Cancer (A) and Advanced Urothelial Carcinoma (B) **eFigure 5.** Cumulative Incidence Function by Sex in Patients With Advanced Urothelial Carcinoma

Supplement 2.
Data Sharing Statement
