# Supplementary material for: Trends and Disparities in Next-Generation Sequencing in Metastatic Prostate and Urothelial Cancers
Source: JAMA Netw Open. 2024 Jul 18;7(7):e2423186. doi: 10.1001/jamanetworkopen.2024.23186 (PMC11258596; doi:10.1001/jamanetworkopen.2024.23186)
Supplement: Supplement 1. — eTable 1. Cumulative Incidence at 6 Months, 1 Year, 2 Years, and 3 Years by Year of Diagnosis and Different Exposures (Race/Ethnicity, Socioeconomic Status, Region, and Insurance) in Patients With Metastatic Prostate Cancer eTable 2. Cumulative Incidence at 6 Months, 1 Year, 2 Years, and 3 Years by Year of Diagnosis and Different Exposures (Race/Ethnicity, Socioeconomic Status, Region, Insurance, and Sex) in Patients With Advanced Urothelial Carcinoma eFigure 1. Cumulative Incidence Function by Race/Ethnicity in Patients With Metastatic Prostate Cancer (A) and Advanced Urothelial Carcinoma (B) eFigure 2. Cumulative Incidence Function by Socioeconomic Status In Patients With Metastatic Prostate Cancer (A) and Advanced Urothelial Carcinoma (B) eFigure 3. Cumulative Incidence Function by Region in Patients With Metastatic Prostate Cancer (A) and Advanced Urothelial Carcinoma (B) eFigure 4. Cumulative Incidence Function by Insurance Plan in Patients With Metastatic Prostate Cancer (A) and Advanced Urothelial Carcinoma (B) eFigure 5. Cumulative Incidence Function by Sex in Patients With Advanced Urothelial Carcinoma [file jamanetwopen-e2423186-s001.pdf]

Supplemental Online Content

Hage Chehade C, Jo Y, Gebrael G, et al. Trends and disparities in next-generation sequencing in metastatic prostate and urothelial cancers. *JAMA Netw Open*. 2024;7(7):e2423186. doi:10.1001/jamanetworkopen.2024.23186

eTable 1. Cumulative Incidence at 6 Months, 1 Year, 2 Years, and 3 Years by Year of Diagnosis and Different Exposures (Race/Ethnicity, Socioeconomic Status, Region, and Insurance) in Patients With Metastatic Prostate Cancer

eTable 2. Cumulative Incidence at 6 Months, 1 Year, 2 Years, and 3 Years by Year of Diagnosis and Different Exposures (Race/Ethnicity, Socioeconomic Status, Region, Insurance, and Sex) in Patients With Advanced Urothelial Carcinoma

eFigure 1. Cumulative Incidence Function by Race/Ethnicity in Patients With Metastatic Prostate Cancer (A) and Advanced Urothelial Carcinoma (B)

eFigure 2. Cumulative Incidence Function by Socioeconomic Status In Patients With Metastatic Prostate Cancer (A) and Advanced Urothelial Carcinoma (B)

eFigure 3. Cumulative Incidence Function by Region in Patients With Metastatic Prostate Cancer (A) and Advanced Urothelial Carcinoma (B)

eFigure 4. Cumulative Incidence Function by Insurance Plan in Patients With Metastatic Prostate Cancer (A) and Advanced Urothelial Carcinoma (B)

eFigure 5. Cumulative Incidence Function by Sex in Patients With Advanced Urothelial Carcinoma

This supplemental material has been provided by the authors to give readers additional information about their work.

© 2024 Hage Chehade C et al. *JAMA Network Open.*

eTable 1. Cumulative Incidence at 6 Months, 1 Year, 2 Years, and 3 Years by Year of Diagnosis and Different Exposures (Race/Ethnicity, Socioeconomic Status, Region, and Insurance) in Patients With Metastatic Prostate Cancer

	mPC (n = 11,927)				
	Cumulative incidence, % (95% CI)				*p*
**Variable**	**At 6-mo**	**At 1-yr**	**At 2-yr**	**At 3-yr**	
**mPC diagnosis year**					
2015	0.8 (0.8-0.8)	1.3 (1.3-1.3)	4 (4-4)	8.5 (8.5-8.5)	
2016	2 (2-2)	3.8 (3.8-3.8)	8.3 (8.3-8.3)	14 (13.9-14)	
2017	5 (5-5)	7.2 (7.2-7.3)	13.7 (13.7-13.8)	19 (19-19.1)	
2018	5.5 (5.5-5.5)	8.4 (8.4-8.5)	17.2 (17.2-17.2)	25.5 (25.4-25.5)	< 0.001
2019	8.9 (8.9-8.9)	14.8 (14.8-14.8)	27.6 (27.6-27.6)	33.8 (33.8-33.9)	
2020	17.4 (17.4-17.4)	23.2 (23.1-23.2)	33.9 (33.9-33.9)	41.2 (41.2-41.3)	
2021	22.2 (22.2-22.2)	28.4 (28.4-28.5)	37 (37-37.1)	-	
2022	26.7 (26.6-26.7)	32.7 (32.6-32.8)	-	-	
**Race / Ethnicity**					
Asian non-Hispanic	7.9 (7.8-8)	11.9 (11.7-12)	18.9 (18.7-19.1)	24 (23.8-24.3)	
Black non-Hispanic	8.5 (8.5-8.5)	11.6 (11.6-11.6)	17.9 (17.9-18)	22.9 (22.8-22.9)	
Hispanic/Latino	8.4 (8.4-8.4)	10 (10-10)	16.2 (16.1-16.2)	21.9 (21.8-21.9)	< 0.001
White non-Hispanic	11.3 (11.3-11.3)	15.2 (15.2-15.2)	23 (23-23.1)	28.7 (28.7-28.7)	
Other[a]	9.4 (9.4-9.4)	13.6 (13.6-13.6)	22.8 (22.7-22.8)	28.9 (28.9-28.9)	
Unknown	12.5 (12.5-12.5)	16.8 (16.8-16.9)	23.9 (23.8-23.9)	29.6 (29.6-29.7)	
**Socioeconomic status**					
5 (Highest)	12.1 (12.1-12.2)	17.1 (17-17.1)	24.1 (24.1-24.1)	30 (30-30)	0.002
4	11.3 (11.3-11.3)	15.1 (15-15.1)	22.5 (22.5-22.5)	28.8 (28.8-28.8)	
3	10.8 (10.8-10.8)	14.1 (14.1-14.1)	22.6 (22.6-22.6)	27.9 (27.9-28)	

© 2024 Hage Chehade C et al. *JAMA Network Open.*

2	10.2 (10.2-10.2)	14 (13.9-14)	21.6 (21.6-21.6)	27.9 (27.8-27.9)	
1 (Lowest)	8.3 (8.3-8.3)	11.4 (11.4-11.4)	18.9 (18.9-18.9)	23 (23-23)	
Unknown	10.1 (10.1-10.1)	13.8 (13.8-13.8)	22.2 (22.2-22.2)	27.8 (27.7-27.8)	
**Region**					
Midwest	10.1 (10.1-10.2)	13.4 (13.4-13.4)	21.4 (21.4-21.4)	26.4 (26.4-26.4)	< 0.001
Northeast	11.1 (11.1-11.1)	14.2 (14.2-14.2)	21.4 (21.4-21.5)	26.6 (26.6-26.6)	
South	9.9 (9.9-9.9)	14 (14-14)	22.3 (22.3-22.3)	28.1 (28.1-28.2)	
West	8.8 (8.8-8.8)	12.3 (12.3-12.3)	18.1 (18.1-18.1)	22.3 (22.3-22.3)	
Unknown	13.7 (13.7-13.7)	17.9 (17.9-17.9)	26.1 (26.1-26.1)	33.3 (33.2-33.3)	
**Insurance**					
Commercial health plan	13.3 (13.3-13.3)	17.8 (17.8-17.8)	25.6 (25.6-25.6)	30.7 (30.7-30.7)	< 0.001
Medicare / other government program	8.9 (8.9-9)	11.6 (11.6-11.6)	19.6 (19.6-19.7)	25.1 (25.1-25.1)	
Medicaid	9.5 (9.4-9.6)	9.5 (9.4-9.6)	14.4 (14.3-14.6)	18.4 (18.2-18.6)	
Others	15.1 (15.1-15.2)	19.7 (19.7-19.8)	28.1 (28.1-28.2)	33.2 (33.1-33.3)	
Unknown	5.9 (5.9-5.9)	9.1 (9.1-9.1)	16.5 (16.5-16.5)	23.4 (23.4-23.5)	

[a]Other race-ethnicity category included Alaska Native, American Indian, Native Hawaiian, other Pacific Islander who are not Hispanic or Latino or a race description which falls in multiple race categories

Abbreviations: CI, confidence interval; mPC, metastatic prostate cancer.

© 2024 Hage Chehade C et al. *JAMA Network Open.*

eTable 2. Cumulative Incidence at 6 Months, 1 Year, 2 Years, and 3 Years by Year of Diagnosis and Different Exposures (Race/Ethnicity, Socioeconomic Status, Region, Insurance, and Sex) in Patients With Advanced Urothelial Carcinoma

	aUC (n = 6.490)				
	Cumulative incidence, % (95% CI)				***p***
**Variable**	**At 6-mo**	**At 1-yr**	**At 2-yr**	**At 3-yr**	
**aUC diagnosis year**					
2015	4.2 (4.2-4.2)	6.9 (6.9-6.9)	10.8 (10.8-10.9)	13.5 (13.4-13.5)	< 0.001
2016	6.5 (6.5-6.6)	9.4 (9.4-9.4)	12 (12-12)	13.7 (13.7-13.8)	
2017	8.4 (8.4-8.4)	12.1 (12.1-12.1)	18.2 (18.2-18.3)	21.3 (21.2-21.3)	
2018	12.9 (12.9-12.9)	18.5 (18.5-18.5)	26.7 (26.6-26.7)	28.1 (28.1-28.2)	
2019	23.7 (23.6-23.7)	30 (30-30.1)	34 (34-34.1)	36.2 (36.2-36.3)	
2020	35 (35-35.1)	41.1 (41-41.1)	45.9 (45.8-46)	47.4 (47.3-47.4)	
2021	39.8 (39.7-39.8)	48.1 (48.1-48.2)	50.9 (50.8-50.9)	-	
2022	47.2 (47.1-47.3)	52.5 (52.4-52.6)	-	-	
**Race / Ethnicity**					
Asian non-Hispanic	23.8 (23.3-24.2)	28 (27.5-28.5)	29.5 (29-30.1)	37 (36.4-37.7)	0.002
Black non-Hispanic	16.3 (16.2-16.4)	20.9 (20.8-21)	24.8 (24.7-24.9)	26.3 (26.1-26.4)	
Hispanic/Latino	17.9 (17.7-18)	22.6 (22.4-22.7)	27.5 (27.3-27.7)	28.1 (28-28.3)	
White non-Hispanic	21.5 (21.5-21.5)	26.8 (26.8-26.8)	31.2 (31.2-31.2)	33.1 (33.1-33.1)	
Other[a]	23.4 (23.3-23.4)	29 (28.9-29)	36.2 (36.1-36.2)	38.9 (38.8-39)	
Unknown	26.7 (26.7-26.8)	30.2 (30.1-30.3)	33.7 (33.6-33.8)	34.9 (34.8-35)	
**Socioeconomic status**					
5 (Highest)	24.2 (24.2-24.2)	30.2 (30.2-30.2)	34 (33.9-34)	36 (36-36)	0.003
4	22.9 (22.9-22.9)	28.1 (28-28.1)	33.3 (33.3-33.4)	35.4 (35.4-35.4)	
3	23.2 (23.2-23.2)	28.2 (28.2-28.2)	33.3 (33.2-33.3)	35.2 (35.1-35.2)	

© 2024 Hage Chehade C et al. *JAMA Network Open.*

2	20.8 (20.7-20.8)	25.7 (25.6-25.7)	29.7 (29.7-29.7)	31.5 (31.5-31.6)	
1 (Lowest)	19.4 (19.4-19.5)	24.1 (24.1-24.2)	28.3 (28.3-28.4)	30 (30-30.1)	
Unknown	18.3 (18.3-18.3)	22.9 (22.8-22.9)	28.5 (28.5-28.6)	30.1 (30-30.2)	
**Region**					
Midwest	15.7 (15.7-15.7)	20.6 (20.5-20.6)	25.9 (25.9-26)	28.4 (28.3-28.4)	
Northeast	22.3 (22.2-22.3)	26.9 (26.8-26.9)	32.3 (32.2-32.3)	34.1 (34-34.2)	
South	24.4 (24.4-24.4)	29.6 (29.6-29.6)	34 (34-34)	36 (35.9-36)	< 0.001
West	20.5 (20.5-20.6)	25.6 (25.5-25.6)	30.5 (30.4-30.5)	32.6 (32.5-32.7)	
Unknown	20.5 (20.5-20.5)	26.2 (26.1-26.2)	30.2 (30.2-30.3)	31.5 (31.5-31.5)	
**Insurance**					
Commercial health plan	25 (25-25)	30.1 (30.1-30.2)	34.6 (34.6-34.6)	36.4 (36.3-36.4)	
Medicare / other government program	18.9 (18.8-18.9)	24.5 (24.4-24.5)	29.9 (29.9-30)	32.4 (32.4-32.5)	< 0.001
Medicaid	14.7 (14.6-14.9)	22.7 (22.4-22.9)	26.3 (26-26.6)	27.4 (27.1-27.7)	
Others	27.5 (27.4-27.6)	32.7 (32.6-32.8)	36.5 (36.4-36.6)	39.2 (39.1-39.3)	
Unknown	14.9 (14.9-14.9)	19.4 (19.4-19.4)	24.2 (24.2-24.2)	25.8 (25.8-25.9)	
**Sex**					
Men	21.6 (21.6-21.6)	26.5 (26.5-26.5)	31.1 (31.1-31.1)	33 (33-33)	0.125
Women	22.8 (22.7-22.8)	28.6 (28.5-28.6)	33.2 (33.2-33.2)	35.2 (35.1-35.2)	

[a]Other race-ethnicity category included Alaska Native, American Indian, Native Hawaiian, other Pacific Islander who are not Hispanic or Latino or a race description which falls in multiple race categories.

Abbreviations: aUC, advanced urothelial carcinoma; CI, confidence interval.

© 2024 Hage Chehade C et al. *JAMA Network Open.*

eFigure 1. Cumulative Incidence Function by Race/Ethnicity in Patients With Metastatic Prostate Cancer (A) and Advanced Urothelial Carcinoma (B)

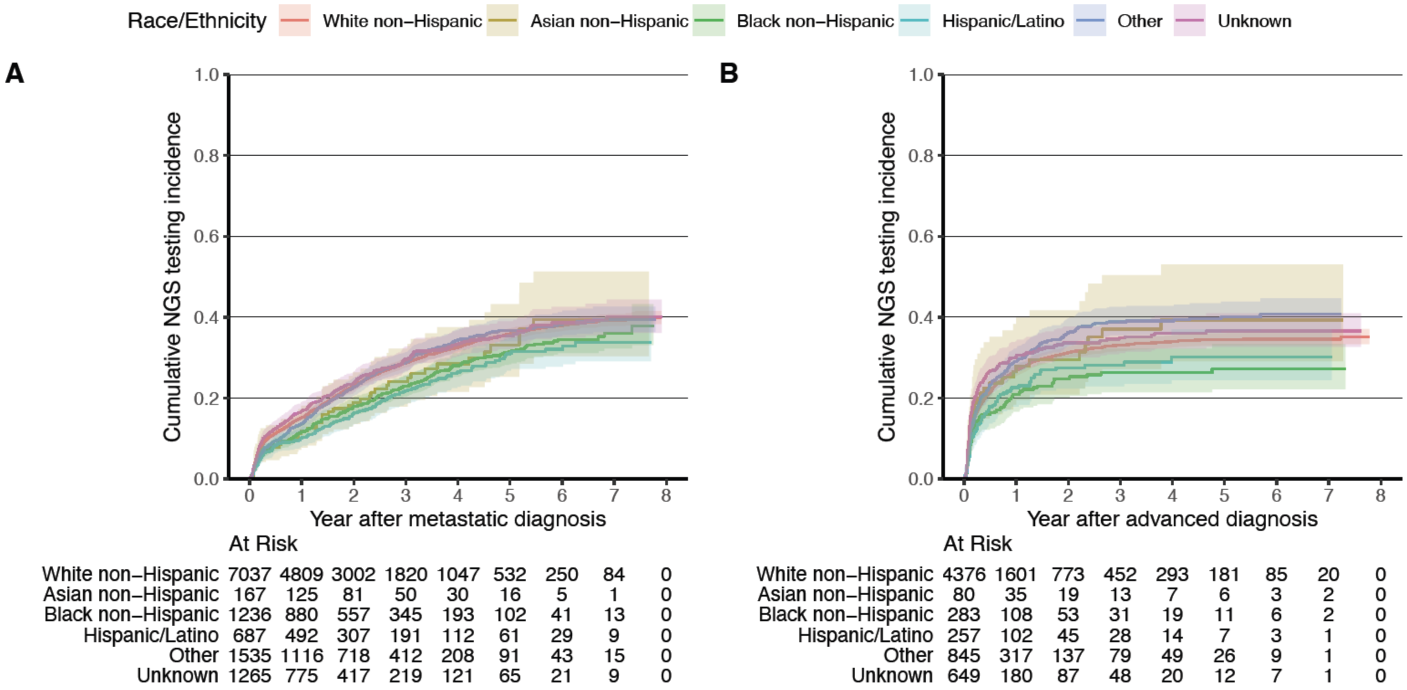

Other race-ethnicity category included Alaska Native, American Indian, Native Hawaiian, other Pacific Islander who are not Hispanic or Latino or a race description which falls in multiple race categories

© 2024 Hage Chehade C et al. *JAMA Network Open.*

eFigure 2. Cumulative Incidence Function by Socioeconomic Status In Patients With Metastatic Prostate Cancer (A) and Advanced Urothelial Carcinoma (B)

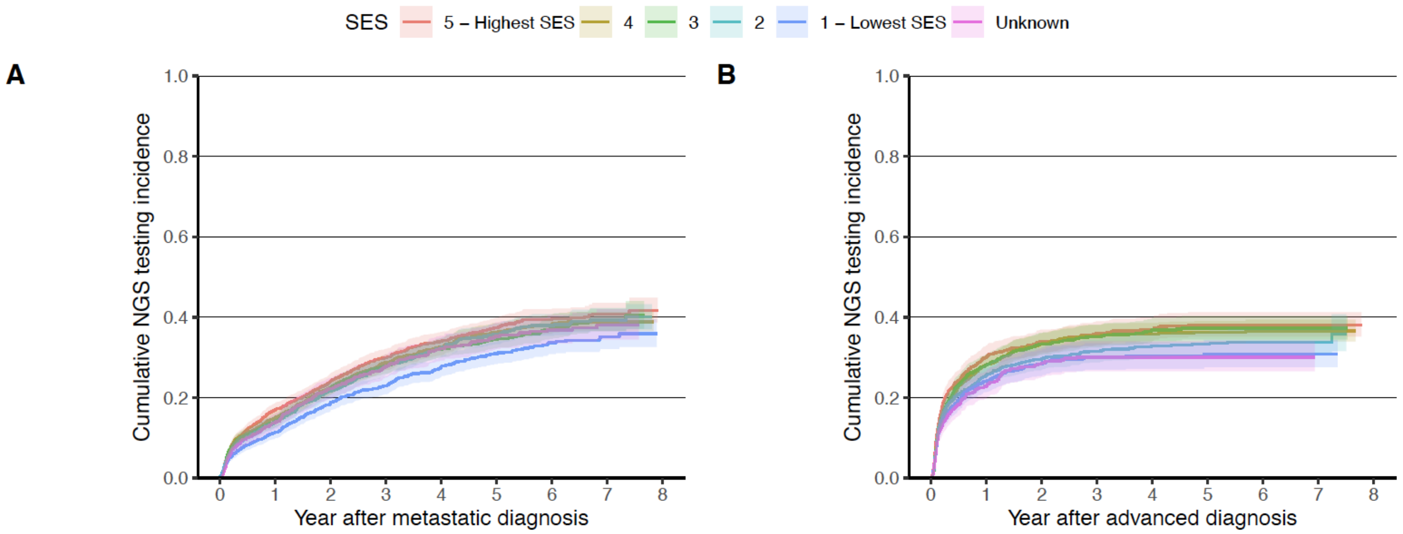

At Risk (A)

	0	1	2	3	4	5	6	7	8
5 – Highest SES	2271	1518	981	589	326	176	76	27	0
4	2580	1790	1125	651	372	182	82	27	0
3	2310	1597	969	594	334	174	71	17	0
2	1983	1379	858	488	281	146	67	29	0
1 – Lowest SES	1663	1153	704	444	250	123	62	20	0
Unknown	1120	760	445	271	148	66	31	11	0

At Risk (B)

	0	1	2	3	4	5	6	7	8
5 – Highest SES	1173	401	206	127	79	49	25	6	0
4	1425	528	259	153	103	67	32	9	0
3	1238	454	214	127	75	43	19	3	0
2	1134	412	202	124	75	50	22	6	0
1 – Lowest SES	880	328	142	73	47	24	12	3	0
Unknown	640	220	91	47	23	10	3	0	0

Abbreviations: NGS, next-generation sequencing; SES, socioeconomic status.

© 2024 Hage Chehade C et al. *JAMA Network Open.*

eFigure 3. Cumulative Incidence Function by Region in Patients With Metastatic Prostate Cancer (A) and Advanced Urothelial Carcinoma (B)

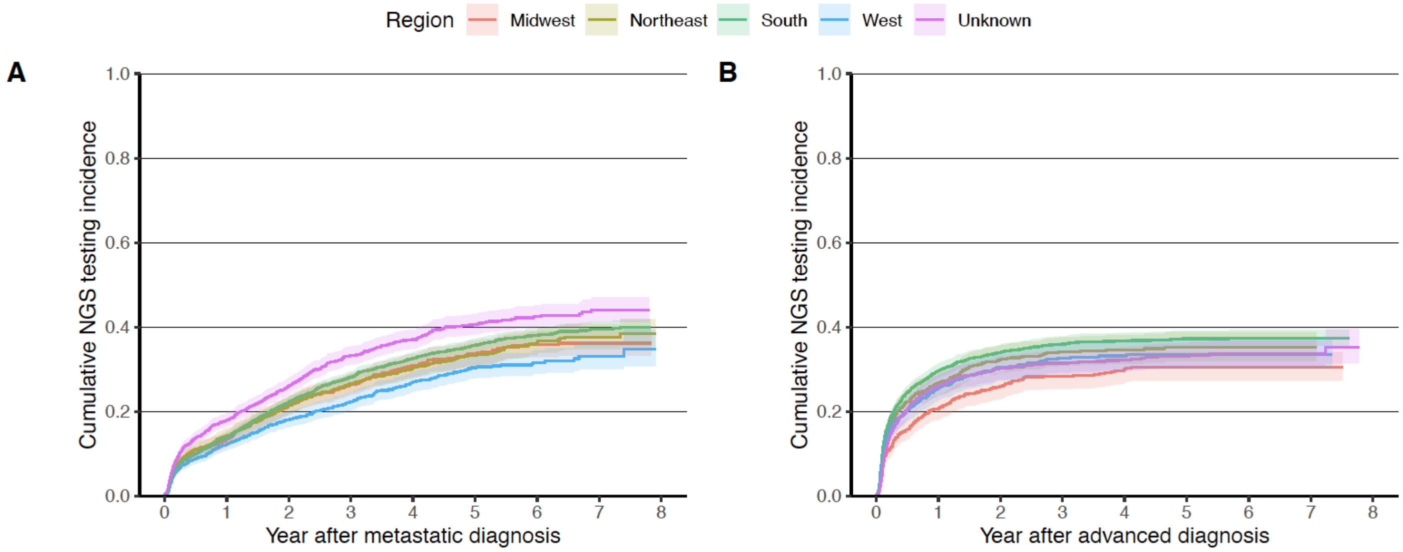

A — At Risk

	0	1	2	3	4	5	6	7	8
Midwest	1287	928	591	367	200	107	45	17	0
Northeast	1710	1203	758	459	259	131	63	21	0
South	4982	3398	2044	1207	697	364	160	57	0
West	1763	1220	773	473	253	105	49	11	0
Unknown	2185	1448	916	531	302	160	72	25	0

B — At Risk

	0	1	2	3	4	5	6	7	8
Midwest	786	321	137	81	51	33	15	5	0
Northeast	869	295	150	85	51	31	14	3	0
South	2882	990	468	277	171	102	45	6	0
West	799	278	123	70	44	30	16	4	0
Unknown	1154	459	236	138	85	47	23	9	0

Abbreviations: NGS, next-generation sequencing.

© 2024 Hage Chehade C et al. *JAMA Network Open.*

eFigure 4. Cumulative Incidence Function by Insurance Plan in Patients With Metastatic Prostate Cancer (A) and Advanced Urothelial Carcinoma (B)

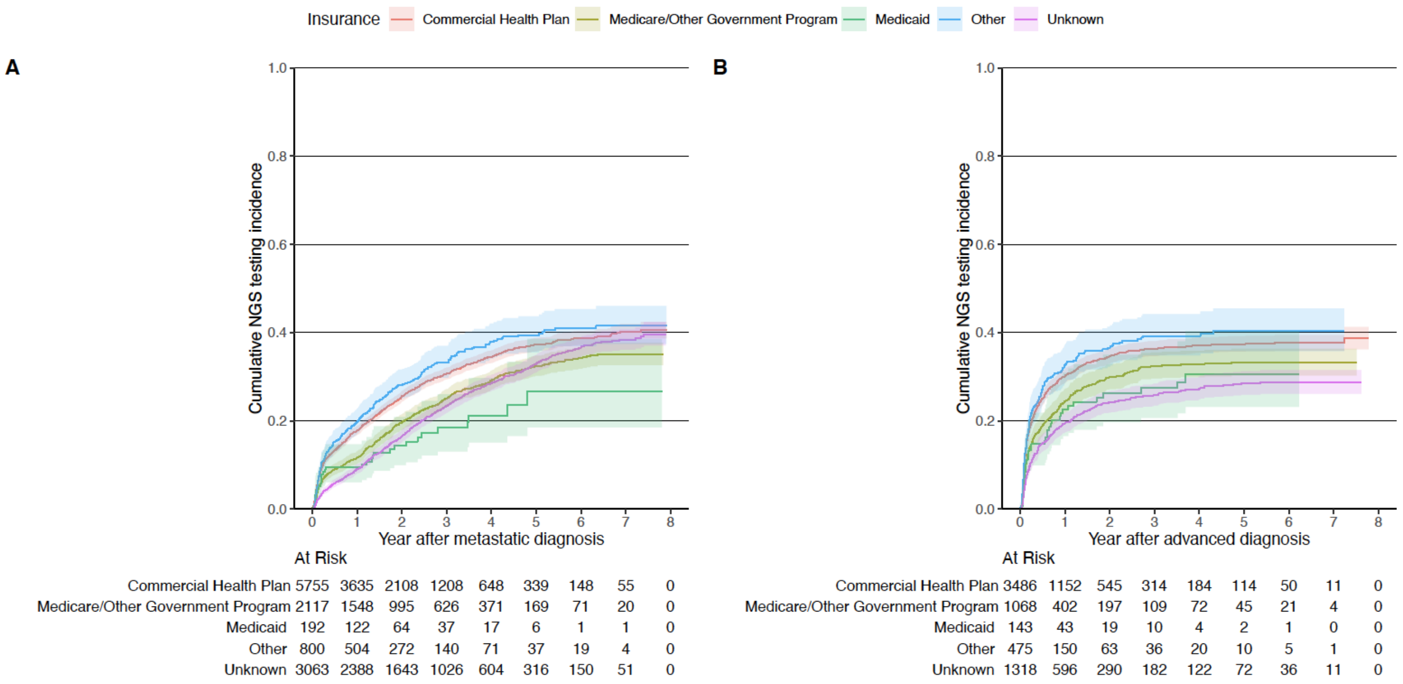

Abbreviations: NGS, next-generation sequencing.

© 2024 Hage Chehade C et al. *JAMA Network Open.*

eFigure 5. Cumulative Incidence Function by Sex in Patients With Advanced Urothelial Carcinoma

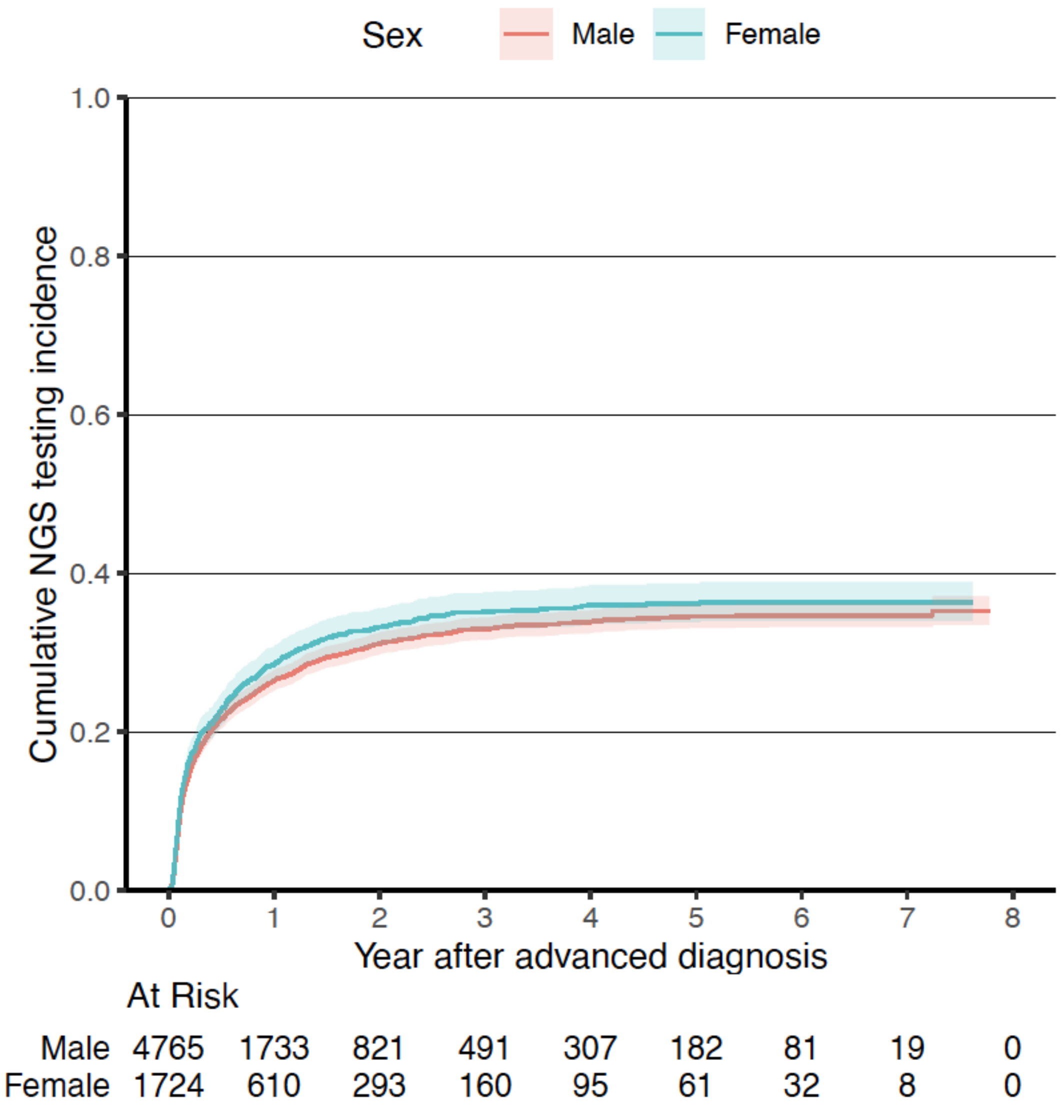

Abbreviations: NGS, next-generation sequencing.

© 2024 Hage Chehade C et al. *JAMA Network Open.*
