# Supplementary material for: Trends and Disparities in Next-Generation Sequencing in Metastatic Prostate and Urothelial Cancers
Source: JAMA Netw Open. 2024 Jul 18;7(7):e2423186. doi: 10.1001/jamanetworkopen.2024.23186 (PMC11258596; doi:10.1001/jamanetworkopen.2024.23186)
Supplement: Supplement 2. — Data Sharing Statement [file jamanetwopen-e2423186-s002.pdf]

Data Sharing Statement

Hage Chehade. Trends and Disparities in Next-Generation Sequencing in Metastatic Prostate and Urothelial Cancers. *JAMA Netw Open*. Published July 18, 2024. doi:10.1001/jamanetworkopen.2024.23186

Data
Data available: No

Additional Information
Explanation for why data not available: The data that support the findings of this study have been originated by Flatiron Health, Inc. Requests for data sharing by license or by permission for the specific purpose of replicating results in this manuscript can be submitted to dataaccess@flatiron.com.
